# Zinc Excess Triggered Polyamines Accumulation in Lettuce Root Metabolome, As Compared to Osmotic Stress under High Salinity

**DOI:** 10.3389/fpls.2016.00842

**Published:** 2016-06-10

**Authors:** Youssef Rouphael, Giuseppe Colla, Letizia Bernardo, David Kane, Marco Trevisan, Luigi Lucini

**Affiliations:** ^1^Department of Agricultural Sciences, University of Naples Federico IINaples, Italy; ^2^Department of Agricultural and Forestry Sciences, University of TusciaViterbo, Italy; ^3^Institute of Environmental and Agricultural Chemistry, Catholic University of the Sacred HeartPiacenza, Italy; ^4^Enviresearch Ltd., Nanotechnology Centre, Newcastle UniversityNewcastle Upon Tyne, UK

**Keywords:** *Lactuca sativa* L., metabolomics, heavy metals, abiotic stress, pathways analysis

## Abstract

Abiotic stresses such as salinity and metal contaminations are the major environmental stresses that adversely affect crop productivity worldwide. Crop responses and tolerance to abiotic stress are complex processes for which “-omic” approaches such as metabolomics is giving us a newest view of biological systems. The aim of the current research was to assess metabolic changes in lettuce (*Lactuca sativa* L.), by specifically probing the root metabolome of plants exposed to elevated isomolar concentrations of NaCl and ZnSO_4_. Most of the metabolites that were differentially accumulated in roots were identified for stress conditions, however the response was more intense in plants exposed to NaCl. Compounds identified in either NaCl or ZnSO_4_ conditions were: carbohydrates, phenolics, hormones, glucosinolates, and lipids. Our findings suggest that osmotic stress and the consequent redox imbalance play a major role in determining lettuce root metabolic response. In addition, it was identified that polyamines and polyamine conjugates were triggered as a specific response to ZnSO_4_. These findings help improve understanding of how plants cope with abiotic stresses. This information can be used to assist decision-making in breeding programs for improving crop tolerance to salinity and heavy metal contaminations.

## Introduction

Farmers around the world only harvest an average of 50% of the potential yield they would obtain under optimal conditions (FAO, 2014). The gap between the actual yield and yield potential, known as “yield gap” is attributable to abiotic factors, especially salinity, drought, and heavy metals contamination (Moshelion and Altman, 2015). Excessive sodium chloride (NaCl) salinity in soil or water (Tester and Davenport, 2003) is a serious threat for agriculture. High concentrations of NaCl can inhibit plant growth and result in decline of productivity. This is due to (i) ionic and osmotic effects on nutritional balance and metabolic process, such as photosynthetic machinery (Parida and Das, 2005), nitrogen assimilation (Lucini et al., 2015), protein synthesis (Giridara Kumar et al., 2003); (ii) induction of the over synthesis of reactive oxygen species (ROS) (Colla et al., 2010).

In addition, heavy metal toxicity is also considered a major abiotic stress affecting worldwide agricultural production (Kumar et al., 2015a,b). Unlike several heavy metals, such as cadmium (Cd), chromium (Cr), lead (Pb), and mercury (Hg), zinc (Zn) is considered an essential microelement for higher plants. Zinc is required at optimal concentration of 20 μg g^−1^ dry weight for the normal functioning of cell metabolism as well as plant growth and development (Broadley et al., 2007; Marschner, 2012). The normal range of Zn in the nutrient solutions for satisfying the crop requirement of Zn is between 0.05 and 0.50 mg/L (Jones, 2005). It is also involved in protein synthesis and many metabolic processes as one of the major cofactors of numerous enzymes (e.g., carbonic anhydrase, Cu/Zn superoxide dismutase, and matrix metalloproteinases; Cakmak, 2000). For most crop species, the critical toxicity concentration in leaf tissue ranges from 100 to >300 μg g^−1^dw, the latter being more typical (Ruano et al., 1988; Marschner, 2012). However, anthropogenic activities such as mining, burning of fossil fuels, and agricultural practices have lead to Zn accumulation in soil (Nagajyoti et al., 2010). High Zn concentrations in soil and water can disturb physiological, biochemical, and metabolic processes leading to stunted plant growth by altering carbohydrate metabolism (Marschner, 2012) and photosynthesis, lowering the concentration of essential nutrients such as magnesium and iron (Sagardoy et al., 2010), causing oxidative damage to membranes, and interfering with DNA replication (Broadley et al., 2007; Vassilev et al., 2007).

Recent research into the development of “omics,” including genomics, ionomics, metabolomics, proteomics, and transcriptomics, has boosted “plant science” and helped clarify the functions of many key genes, proteins, and metabolite networks involved in plant responses under unfavorable soil and environmental conditions (Rodziewicz et al., 2014). The field of metabolomics has grown substantially over the past decade and has proven to be an important and efficient tool in plant responses to abiotic stresses (Rodziewicz et al., 2014) that allows the identification of potential biomarkers linked to improved stress tolerance. This can then lead to efficient genetic improvement programs (Weckwerth and Kahl, 2013). Knowledge of root molecular changes in response to stress is fundamental since roots are highly sensitive to several types of abiotic stresses (Jiang et al., 2007). Although plant response to salt stress has been only investigated in vegetables at leaf metabolic level (Lucini et al., 2015), to our knowledge no published data is available related to root metabolomic responses to Zn exposure. As a large amount of information has been published concerning soil salinity, this study considered NaCl as a positive control, able to exert an iso-osmotic stress similar to ZnSO_4_.

The objective of the present study was to elucidate the metabolic changes in lettuce (*Lactuca sativa* L.), by probing the root metabolome of plants exposed to elevated concentrations of NaCl and ZnSO_4_.

## Materials and methods

### Plant material, growth conditions, and treatments

Lettuce (*L. sativa* L. var. *longifolia* cv “Bionda degli Ortolani”) seedlings purchased from a local nursery were transplanted at the three true-leaves stage, into plastic pots (diameter 16 cm) filled with commercial peat-based substrate having pH 6.2 in water, electrical conductivity 0.25 dS/m, porosity 92% v/v, CSC 98 meq/100 g, 140 mg/L N, 43 mg/L P, 150 mg/L K, 150 mg/L Ca, 50 mg/L Mg, 2.5 mg/L Fe, 1.3 mg/L Mn, 0.1 mg/L Zn. Lettuce plants were cultivated under controlled environment (average air temperature of 20°C). Treatments were arranged in a randomized complete block design with 10 pots per treatment, each of them having four plants. The pot experiment consisted of three nutrient solution treatments: a non-salt control, 100 mM NaCl and 100 mM ZnSO_4_ as described by Lucini and Bernardo (2015). The study was performed in terms of equimolar concentrations of the two different salts in order to evaluate the ion effects of Na and Zn on root metabolic profiling. The NaCl and ZnSO_4_ nutrient solutions were applied 1 week after transplanting. Pots were watered as required by the crop (3–5 irrigations per week); all treatments received the same watering regime. At the end of the experiment (37 days after transplanting) leaves were collected, fresh tissue biomass measured and samples were then quenched in liquid nitrogen and grounded into a fine powder using mortar and pestle.

### Mineral analysis

The root tissues were analyzed for the following elements: K, Mg, Fe, Na, and Zn. Potassium and Mg were selected for their antagonism with Na for root uptake while Fe was selected for its antagonistic interaction with Zn. A portion (250 mg) of each sample was mineralized in 0.5 mL of hydrogen peroxide (H_2_O_2_) and 1 mL of nitric acid (HNO_3_) for 6 h, diluted in 5% nitric acid and analyzed by inductively coupled plasma atomic emission spectroscopy (ICP Iris; Thermo Optek, Milano, Italy; Karla, 1998). A multi-element source was used for calibration purposes and a certified reference material was analyzed prior to sample analyses. Each sample was read in triplicate.

### Metabolomic analysis

The untargeted screening of plant metabolites was carried out through UHPLC chromatographic coupled to a hybrid quadrupole-time-of-flight mass spectrometer (UHPLC/Q-TOF). An Agilent 1290 UHPLC liquid chromatograph was coupled to an Agilent G6550 mass spectrometer detector (Agilent Technologies Santa Clara, CA, USA) through a Dual Electrospray JetStream ionization system.

Reverse phase chromatography was carried out on an Agilent Zorbax Eclipse-plus column (75 × 2.1 mm i.d., 1.8 μm). The LC mobile phase consisted of water (A) and methanol (B), flowing at 220 μL min^−1^ and 35°C. Formic acid 0.1% (v/v) and ammonium formate (5 mM) (both from Sigma Aldrich, St. Louis, MO, USA) were added to A and B. The gradient started with 5% B and increased to 90% B within 30 min, to be held for 5 min. The injection volume was 3 μL and mass spectrometer was operated in the positive scan mode to acquire spectra in the range of 100–1600 m/z. Regarding source conditions, sheath gas was nitrogen at 10 L min^−1^ and 350°C, drying gas was nitrogen at 10 L min^−1^ and 280°C, nebulizer pressure was 60 psig, nozzle voltage was 300 V and capillary voltage was 3.5 kV. Lock masses (m/z 121.0509 and 922.0098) were continuously infused in the ionization source by a quaternary pump through a dedicated electrospray.

Raw data were processed through a recursive analysis workflow, using Profinder B.05 (from Agilent Technologies) for feature alignment and filtering after initial deconvolution. Compounds identification was carried out using the whole isotopic pattern of deconvoluted features, hence taking into account accurate mass, isotope accurate spacing, and ratio.

Those compounds that were not present in 100% of the replicates within at least one treatment were discarded. Filtered features were next identified using the database exported from PlantCyc 9.5 (Plant Metabolic Network, http://www.plantcyc.org; released November 2014), using a mass accuracy tolerance threshold of 5 ppm. The peak volume of each compound identified with a score above 80/100 was extracted from the total ions current and exported for statistics and data interpretation. Additionally, the database was integrated using the software Pathway-to-PCDL (Agilent technologies), to create a compounds database (defined as “Personal Compounds Database Library” in the software tool) from the plant pathways available via KEGG (Kanehisa et al., 2016). In this way, annotated compounds could be identified, thus allowing pathway analysis from differential metabolites by using their KEGG ID.

### Statistical analysis

Interpretation of metabolomic results was carried out after recursive identification, using Mass Profiler Professional B.12.05 (Agilent technologies). The identified compounds were log_2_ normalized and abundances baselined against the median of each compound in all samples. Statistics and chemometrics included analysis of variance (ANOVA at *P* = 0.001, Bonferroni multiple testing correction) and fold-change analysis (cut-off = 5), which were combined into volcano plots. Differential metabolites gained from the KEGG-derived database and selected through Volcano analysis were then used to perform pathway analysis using KEGG annotations to link metabolites and metabolic pathways.

Multivariate statistics were also carried out: Unsupervised cluster analysis (hierarchical cluster algorithm; similarity measure set to Euclidean) and Partial Least Squares Discriminant Analysis (PLS-DA; N-fold validation, *N* = 4) were performed. Concerning PLS-DA, the loadings (compounds) used to build the class prediction model were plotted according to their weight within the latent vectors, and those being most relevant in class prediction (i.e., those having a score of above +0.05 rather than below −0.05) were exported to support or complement information from Volcano analysis.

## Results

### Plant biomass and mineral composition

The lettuce biomass decreased in response to an increase in sodium chloride and zinc sulfate concentration in the nutrient solution with more detrimental effects resulting from the NaCl treatment. The percentage reduction in biomass production in comparison to the non-salt control was significantly lower in plants treated with ZnSO_4_ (−25%) than in plants treated with NaCl (−53%, data not shown).

The macro and microelements concentration determined by ICP spectroscopy as a function of the nutrient solution treatments are displayed in Table 1. Sodium and zinc concentrations sharply increased in the corresponding treatments (NaCl and ZnSO_4_, respectively), and the effect was more marked for zinc, which increased more than 16 fold, compared to a < 5-fold increase for sodium. Moreover, NaCl treatment decreased the lettuce root content of K while Fe concentration in root was reduced by ZnSO_4_ treatment.

**Table 1:** **Effects of solution treatments on lettuce root concentration of K, Mg, Na, Fe, and Zn**.

**Treatments**	**K**	**Mg**	**Na**	**Fe**	**Zn**
Control	48.1^b^	1.2^b^	0.67^b^	44.0^a^	40.3^c^
NaCl	46.5^c^	1.9^a^	2.72^a^	44.0^a^	52.3^b^
ZnSO_4_	51.0^a^	1.1^c^	0.59^c^	36.0^b^	674.5^a^
Significance	^**^	^**^	^***^	^**^	^***^

K and Mg values are reported in g kg^-1^, while values of Na, Fe, and Zn are expressed as mg kg^-1^. Superscript letters denote Student-Newman-Keuls post-hoc classes from one-way ANOVA (p = 0.01). Significant at

** P ≤ 0.01 and

*** *P ≤ 0.001*.

### Metabolic profile

Metabolomics in crop science are supposed to be prone to false positives, because databases are poorer than for model plants, and because they lack of tandem MS information. The combination of comprehensive databases like PlantCyc and KEGG in combination with proper data handling (i.e., the use of whole isotopic profile for identification, compounds alignments and filtering, and then recursive analysis) was effective in providing a wide metabolomic dataset and limiting false positives. All compounds were detected with low mass error (nominally below 5 ppm, and in the sub-ppm range in several cases). Starting from a total of more than 2400 compounds detected, recursive analysis and the following filters in Mass Profiler Professional dramatically reduced the compounds in the dataset to a number slightly above a thousand. The whole dataset, as gained after data processing, is provided as Supplementary Material.

**Figure 1:**
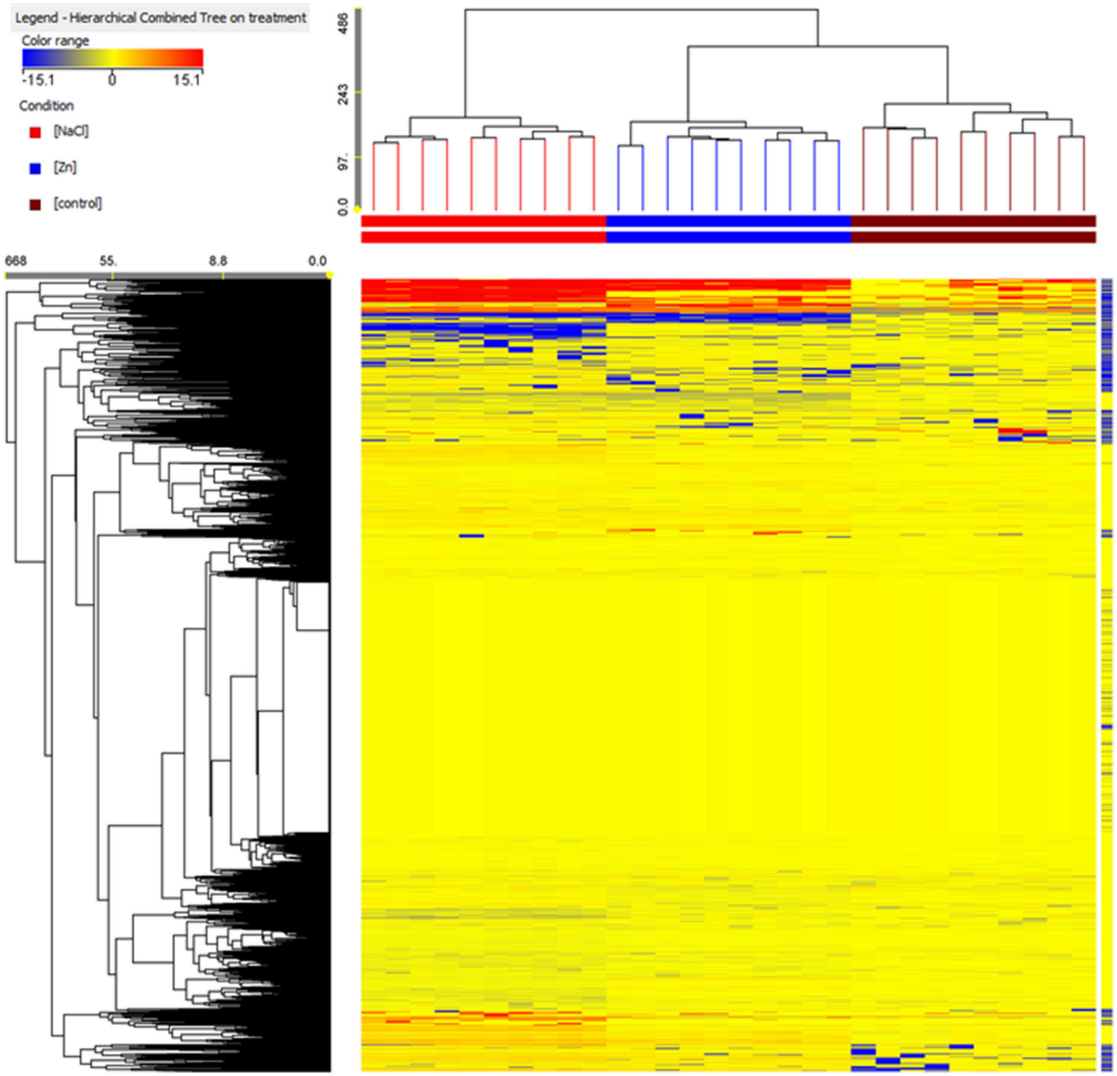
**Not averaged unsupervised cluster analysis on metabolite profile in lettuce roots under NaCl or ZnSO_4_ treatment, as compared to control (Similarity, Euclidean; linkage rule, Ward)**. Compounds intensity was used to build up heat maps, on the basis of which the clusters were generated.

**Figure 2:**
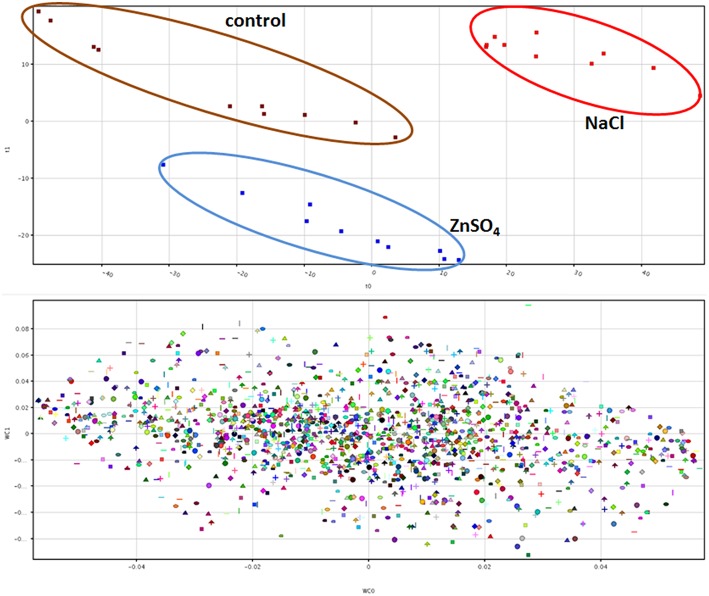
**Partial Least Squares Discriminant analysis (PLS-DA) on lettuce metabolomic profile according to NaCl or ZnSO_4_ stress**. Individual replications are given in the predictive model hyperspace (**upper panel**), while compound loadings view is provided in **lower panel**, where model class prediction scores are plotted.

Differential compounds were selected through Volcano analysis, where analysis of variance and fold-change were combined (using a fold-change cut off = 5 and a *p*-value of 0.001). Unsupervised cluster analysis (Figure 1), carried out from fold-change patterns (heat maps), and resulted in the definition of three well distinct clusters, one per treatment. Consistently, PLS-DA class prediction models gave accurate results (prediction model overall accuracy = 100% after N-fold validation with *N* = 4), in which replications clustered each other within a treatment (Figure 2). Considering that PLS-DA could clearly differentiate the treatments, those compounds being differential (i.e., hose having the highest weight in the class prediction model) were exported from the compound loading view of PLS-DA and pooled with other differential metabolites. The majority of these differential metabolites had already been selected through Volcano analysis thus strengthening differences at metabolome level also through multivariate statistics.

The compounds selected from Volcano analysis and PLS-DA scores are summarized in Tables 2, 3 (ZnSO_4_ treatment against control, and NaCl treatment against control, respectively).

**Table 2:** **Output of Volcano analysis: metabolites resulting differential in lettuce root exposed to zinc stress against control, grouped according to functional classes**.

**Metabolites**		***p* (Corr)**	**FC (abs)**	**Regulation**
Carbohydrates	2-amino-3,7-dideoxy-D-threo-hept-6-ulosonate	7.12E-15	138,963	Down
	1-kestotriose^a^	2.37E-15	13,856,115	Down
	1,1-kestotetraose^a^	8.18E-08	18,210,966	Down
	Maltoheptaose^a^	6.66E-23	23,748,209	Down
	β-D-apiofuranosyl-(1-6)-D-glucose	4.54E-09	10,028,662	Down
	D-hexose –phosphate^a^	0	16	Down
Lipids	Digitoxigenin 3-O-beta-D-quinovoside	7.60E-09	11,217,574	Down
	Eicosapentaenoyl-CoA	6.47E-27	21,449,984	Down
	Solasodine 3-O-beta-D-glucopyranoside	1.38E-09	8,561,974	Down
	Hexadecanedioyl-CoA	3.20E-23	27,931,486	Down
	CDP-1-18:1(9Z)-2-16:0-glycerol^a^	8.77E-07	70,597,266	Down
	Dihomo gamma-linolenoyl-2-enoyl-CoA	0	16	Up
	Phosphoryl-ethanolamine^a^	0	16	Up
	Acetoacetate	0	16	Up
	CDP-1-18:1(9Z)-2-18:1(9Z)-glycerol^a^	1.05E-06	7,139,225	Down
Phenolics	8C-glucosyl-2,5,7-trihydroxyflavanone	5.55E-08	9,611,869	Down
	Isovitexin-7-O-xyloside	8.55E-15	11,610,447	Down
	Afrormosin-7-O-glucoside-6″-O-malonate	1.28E-14	12,522,652	Down
	Pelargonidin 3,7-di-O-beta;-D-glucoside	0	16	Up
	Apigenin-7-O-gentiobioside	0	16	Down
	Catechin^b^	0	16	Up
Oxidative stress	4,9,13-trimethyltetradeca-2,4,6,8,10,12-hexaene-1,14-dial	0	16	Up
	4-trans-3-oxo-undecenoyl-CoA	9.86E-29	23,782,549	Down
Hormones	Gibberellin A34-catabolite^a^	2.27E-11	7,507,979	Down
	Trans-zeatinribosidediphosphate^a^	0	16	Down
Glucosinolates	4-sinapoyloxybutylglucosinolate	7.12E-15	57,585,897	Down
	Benzyl-desulfoglucosinolate	1.24E-04	11,503,575	Up
	(E)-indol-3-ylacetaldoxime	1.53E-17	25,664,116	Down
	Indole-3-acetohydroximoyl-cysteinylglycine	0	16	Up
	Indolepropanolphosphate	0	24,768,307	Up
Phytoalexins	Zealexin B1^a^	1.72E-07	700,998	Up
Alkaloids	Cyclo-dopaglucuronylglucoside	4.54E-05	43,532,617	Up
	Gamma-coniceine	8.00E-30	13,491,078	Up
	S-canadine	3.27E-04	27,555,862	Down
Others	L-carnitine	4.95E-04	863,446	Up
	Pyridoxamine	1.38E-13	18,502,228	Down
	D-myo-inositol (1,2,3,4,6)-pentaikisphosphate^a^	0	16	Down
	Alpha-ethyl-L-glutamate^a^	3.28E-09	17,081,303	Down
	2′-deoxyadenosine	4.74E-04	11,663,641	Down
	4-acetamidobutanal	3.72E-04	8,858,312	Up
	D-xylonolactone	0	22,147,355	Up
	Dihydroxyferuoyl-sinapoyl-spermidine^a^	0	16	Up
	Spermidine^b^	0	16	Up
	5′-Methylthioadenosine^b^	1.75E+02	10,369,577	Down
	S-Adenosyl-L-homocysteine^b^	7.63E+03	16	Up
	Pheophorbide a^b^	5.28E-03	17,494,915	Up

*For each compound, unpaired t-test p-values (Bonferroni multiple testing correction), absolute fold-change (FC) and regulation are provided. According to Mass Profiler Professional output, values of p (Corr) = 0 or FC = 16 denote very high significance at t-test or extremely high fold-change, respectively*.

a *Confirmed by partial least squares discriminant analysis (PLS-DA)*.

b *Resulted from KEGG database using the Pathway-to-PCDL tool*.

**Table 3:** **Output of Volcano analysis: metabolites resulting differential in lettuce root exposed to NaCl stress against control, grouped according to functional classes**.

**Metabolites**		***p* (Corr)**	**FC (abs)**	**Regulation**
Carbohydrates	2-amino-3,7-dideoxy-D-threo-hept-6-ulosonate	5.11E-15	16,183,598	Down
	6-phospho-β-D-glucosyl-(1,4)-D-glucose^a^	0	16	Down
	N-acetyl-beta-glucosaminylamine	2.49E-11	8,647,426	Up
Lipids	L-1-glycero-3-phosphocholine	4.34E-13	26,339,834	Up
	(2S,5R)-2-(2-hydroxypropan-2-yl)-5,9-dimethyl-1-oxaspiro[5.5]undec-8-ene-7,10-dione	2.07E-05	5,876,433	Up
	Dihomo gamma-linolenoyl-2-enoyl-CoA	0	16	Up
	Phosphoryl-ethanolamine^a^	0	16	Up
	Acetoacetate	0	16	Up
	4-alpha-carboxy-ergosta-7,24(24′)-dien-3-beta-ol	0	16	Down
	Leukotriene-C4	2.31E-08	3,415,718	Down
Phenolics	Baicalin	0	31,623,483	Down
	Isovitexin-7-O-xyloside	7.18E-17	19,908,253	Down
	(+)-pisatin	3.05E-19	8,346,783	Up
	Afrormosin-7-O-glucoside-6″-O-malonate^a^	8.86E-16	17,976,381	Down
	(−)-Sativan^a^	6.00E-06	55,322,294	Up
	(−)-Secoisolariciresinol	2.12E-06	60,151,567	Up
	7-Hydroxyflavone	2.50E-08	76,273,413	Up
	Pelargonidin 3,7-di-O-β-D-glucoside	0	16	Up
	Luteolin 7-O-β-D-glucuronide	0	16	Down
	(S)-dihydrodaidzein	1.03E-08	64,842,844	Up
Oxidative stress	Homoglutathione	6.59E-05	58,197,466	Up
	4,9,13-trimethyltetradeca-2,4,6,8,10,12-hexaene-1,14-dial	0	16	Up
	2-carboxy-L-xylonolactone^a^	0	16	Up
	4-O-oxalyl-L-threonate	0	16	Up
	2,3-dioxo-L-gulonate^a^	0	16	Down
	4,4′-diapo-carotene	1.20E-04	29,445,051	Down
Hormones	16,17-dihydro-16-alpha,17-dihydroxy gibberellin A4^a^	3.14E-05	35,296,392	Up
	Gibberellin A98	1.00E-06	55,527,987	Up
Glucosinolates	4-methoxy-3-indolylmethylisothiocyanate^a^	1.37E-27	18,197,252	Up
	4-methylthiobutyl glucosinolate^a^	7.39E-04	12,715,052	Up
	2-hydroxy-3-butenylglucosinolate	7.18E-33	32,002,068	Up
	Benzyl-desulfoglucosinolate^a^	4.53E-05	14,339,488	Up
	Indole-3-acetohydroximoyl-glutathione^a^	1.15E-09	6,475,546	Up
	Indole-3-acetohydroximoyl-cysteinylglycine	0	16	Up
	9-methylthiononylhydroximate	0	16	Down
Phytoalexins and benzoxazinoids	Camalexin	3.69E-27	16,345,359	Up
	(−)-medicarpin	5.59E-08	68,371,882	Up
	Zealexin B1^a^	5.36E-13	1,533,667	Up
	Zealexin A3^a^	6.50E-05	63,843,145	Up
	Zealexin A1	9.87E-15	1,438,294	Up
	TRIBOA- β-D-glucoside	0	16	Down
Terpenes	β-fenchocamphorone	5.55E-06	6,957,294	Down
	(−)-(4S)-α-terpineol	6.23E-12	804,632	Up
Others	Hydroxymethylbilane	1.68E-05	55,967,686	Up
	Porphyrin-ring	0	16	Down
	7,8-dihydropteroate^a^	2.50E-05	5,475,373	Down
	Serotonin	1.13E-05	7,589,837	Up
	Pyridoxamine^a^	3.18E-11	3,496,212	Down
	Phytanoyl-CoA	1.49E-23	1,614,294	Up
	(2S)-2-isopropyl-3-oxosuccinate	3.00E-07	74,671,884	Down
	Shikimate^a^	1.38E-07	57,079,825	Down
	5-O-(indol-3-ylacetyl-myo-inositol) D-galactoside	1.08E-05	50,891,047	Up
	L-cystine^a^	2.65E-29	33,417,527	Up
	(E)-indol-3-ylacetaldoxime^a^	7.72E-20	38,752,243	Down
	Gamma-glutamyl-isopropylamide	1.84E-07	5,467,311	Down
	NADH^a^	2.64E-25	15,208,814	Up
	Dopaminequinone	5.25E-05	2,204,173	Up
	Homoarginine^a^	3.11E-10	50,679,507	Up
	THF-L-glutamate	4.53E-05	5,023,071	Up
	Isoleucinetetrazole	0	24,584,309	Down
	O-succinyl-L-homoserine^a^	0	16	Down

*For each compound, unpaired t-test p-values (Bonferroni multiple testing correction), absolute fold-change (FC) and regulation are provided. According to Mass Profiler Professional output, values of p (Corr) = 0 or FC = 16 denote very high significance at t-test or extremely high fold-change, respectively*.

a *Confirmed by partial least squares discriminant analysis (PLS-DA)*.

A decrease in several carbohydrates could be observed in both treatments, although the decline of fructans (1-kestotriose, 1,1-kestotetraose, and maltoheptaose) was exclusive to zinc stressed roots. However, phytoalexins increased markedly under high salinity than under Zn stress. In more detail Zealexin B1 was significantly found in ZnSO_4_, whereas camalexin, medicarpin, and three different zealexins were found in NaCl; furthermore, Zealexin B1 was much higher (FC 15.34) in NaCl than in ZnSO_4_ treatment (Tables 2, 3). Phenolic compounds were also triggered by both treatments, but not in an unequivocal way; flavones (isovitexin-7-*O*-xyloside, baicalin, scutellarin, and 7-hydroxyflavone), isoflavonoids (sativan, pisatin, and afrormosin derivatives), apigenin-7-*O*-gentiobioside, pelargonidin-3,7-diglucoside, and the lignan secoisolariciresinol, were the differential phenolic compounds. Furthermore, several lipids, particularly those related to membrane (e.g., phosphoryl-ethanolamine and glicero-3-phosphocholine) rather than carotenoids cleavage (4,9,13-trimethyltetradeca-2,4,6,8,10,12-hexaene-1,14-dial) were increased by both stresses (Tables 2, 3). Finally, hormones (gibberellins and a cytokinin), and several glucosinolates and glucosinolate biosynthetic intermediates, were also affected by the treatments, although in an unclear manner.

NaCl stress, despite being osmolar to Zn stress, triggered a more pronounced metabolic response, both in terms of fold-change values and as number of differential metabolites (Tables 2, 3). The volcano analysis for Zn-stressed vs. NaCl-stressed plants is given in detail as Supplementary Table. Ascorbate (2-carboxy-*L*-xylonolactone and 4-*O*-oxalyl-*L*-threonate) and photosynthetic pigments (porphyrin ring and hydroxymethylbilane) degradation products were increased by NaCl stress, as well as homoglutathione. Analogously, phytoalexins (camalexin, medicarpin, zealexins, and TRIBOA-glucoside) were mainly related to NaCl stress conditions (Table 3).

However, alkaloids (γ-coniceine, *S*-canadine, and cyclo-dopaglucuronylglucoside), polyamines (spermidine and spermidine conjugates), and polyamine degradation products (4-acetamidobutanal) were induced only by Zn stress (Table 2).

## Discussion

Root is the first organ feeling abiotic stresses in soil such as salinity and heavy metal contamination (Jiang et al., 2007), thus conditioning stress sensitivity and limiting plant biomass production (Steppuhn and Raney, 2005; Paradisone et al., 2015). Moreover, high Na concentrations in the soil or pore water may depress nutrient-ion activities and produce an extreme ratio of Na/K (Grattan and Grieve, 1999), whereas high zinc concentrations may induce deficiency of Mg and Fe because of the similar ion radius of Zn^2+^ and Fe^2+^ (Sagardoy et al., 2010), and Zn^2+^ and Mg^2+^ (Boardman and McGuire, 1990). Consistently, biomass reduction and imbalance of mineral nutrients were observed in our study. Indeed, the lowest K concentration was observed with the NaCl treatment, whereas the lowest Mg and Fe concentrations were recorded with the ZnSO_4_ treatment (Table 1).

As the pots were regularly watered with NaCl or ZnSO_4_ enriched water solutions during the whole test period, we expect to have gained a steady state regarding the ions available in the substrate, after an initial transition period. Drainage of at least 50% of irrigation volume was achieved to avoid accumulation of salts in the substrate. At this steady state, the Zn^2+^ and Na^+^ concentrations in substrate solution were expected to be comparable and the plants should have been exposed to a similar osmotic stress.

Taking into account that metabolic changes induced by salinity have been widely reported in literature, our interpretation was mainly focused on biochemical changes related to zinc stress. In this view, salinity has been used as a positive control that exerted a similar osmotic stress to zinc sulfate treatment. As a general consideration for the differential metabolites, several compounds were identified in both ZnSO_4_ and NaCl treated plants, with the latter inducing a more pronounced response to stress. The main classes of differential compounds were: carbohydrates, phenolics, hormones (gibberellins a cytokinin), glucosinolates and lipids. Salinity also induced changes in ascorbate and other compounds related to oxidative stress metabolism, together with phytoalexins and terpenes. However, zinc treatment also affected alkaloids and the polyamine profile in the roots (Table 2).

The reduction of carbohydrates is in agreement with other studies, where a decrease in the carbohydrate route of photosynthetic metabolism was associated to Zn stress (Chernyad'ev and Monakhova, 2006). In addition the increase in glucosinolates, traditionally linked to biotic stress, has been reported in heavy metals stressed plants (Poschenrieder et al., 2006) and even more in hyperaccumulator plant species (Asad et al., 2013). Phytoalexins, a class of compounds usually linked to biotic stress, have also been to abiotic stress (Jeandet et al., 2014). Therefore, their involvement in NaCl stress response, although less expected, can be justified.

Osmotic stress is known to induce an oxidative secondary stress that can explain the increase in ascorbate breakdown products and in homoglutathione, which was observed in NaCl stressed roots. Sakuraba et al. (2014) have also reported that chlorophyll breakdown occurs during stress-induced yellowing, according to our findings in NaCl stressed roots. In previous work, on the use of a protein hydrolysate biostimulant on NaCl stressed lettuce, Lucini et al. (2015) achieved comparable results, reporting the involvement of glucosinolates, terpenes, homoglutathione, hormones, and carbohydrates in response to salinity stress.

**Figure 3:**
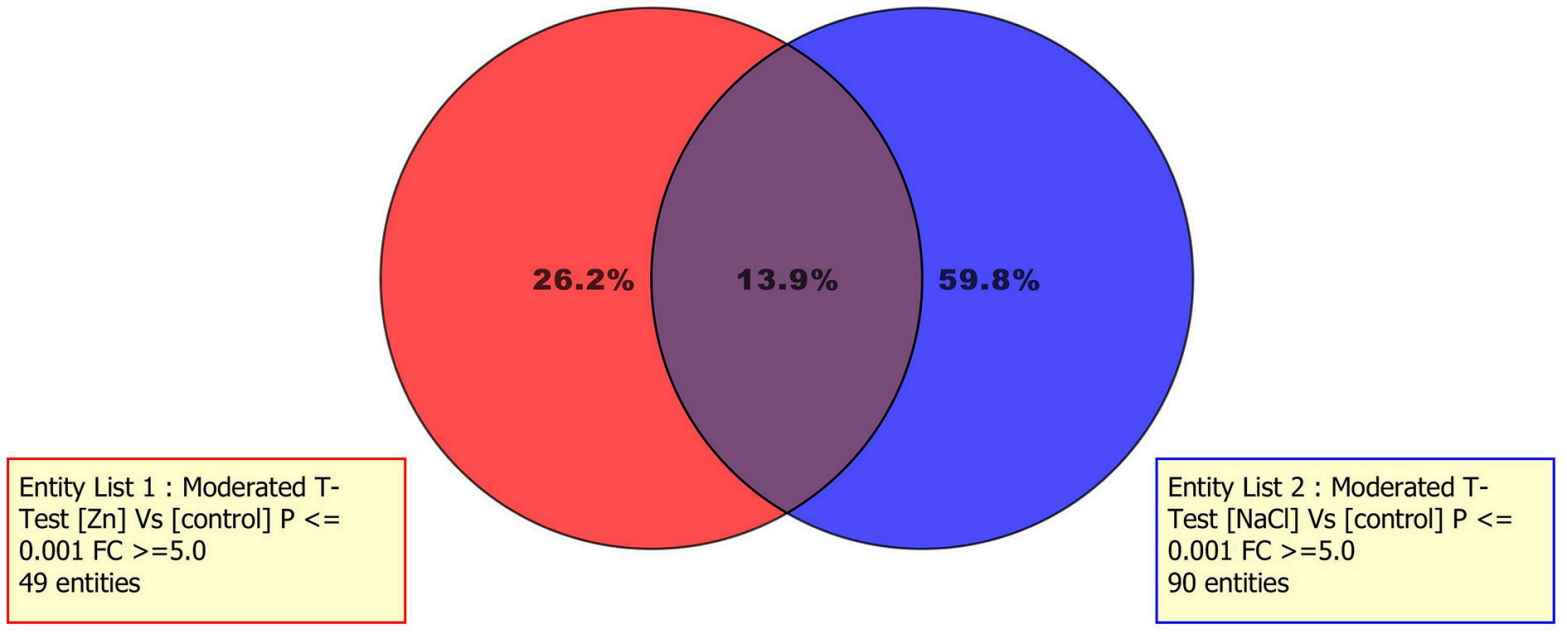
**Venn analysis from metabolites resulting differential after Volcano analysis under either ZnSO_4_ or NaCl stressed plants**.

As a large number of differential metabolites are common to both stresses (Figure 3, Venn analysis from differential metabolites under either Zn excess or salinity stress), it can be postulated that osmotic/oxidative stress is a process influencing lettuce roots following exposure to ZnSO_4_, in agreement with previous results achieved through proteomics using the same experimental design (Lucini and Bernardo, 2015). Furthermore, a larger number of differential metabolites could be evidenced under NaCl stress, suggesting this treatment altered lettuce metabolism in a way stronger than Zn.

The differential metabolites triggered by Zn stress were linked to KEGG metabolic pathways using their KEGG ID (where available via the Pathway-to-PCDL software), in order to drive and support the involvement of specific physiological responses during results interpretation. This analysis highlighted the involvement of the super pathway of polyamine biosynthesis (two matched entities: spermidine and 5-mehylthioadenosine; *p* < 0.001), spermidine hydroxycinnamic acid conjugate biosynthesis (two matched entities: spermidine and S-adenosyl-L-homocysteine; *p* < 0.001), chlorophyll a degradation (one matched entity: pheophorbide a; *p* < 0.001), and 2,3-flavanols biosynthesis (one matched entity: catechin; *p* < 0.001). Therefore, together with chlorophyll degradation and flavonols biosynthesis, the pathway analysis clearly confirmed the specific involvement of polyamine metabolism, and spermidine in particular, to ameliorate disorders from zinc stress. To provide further confidence regarding the involvement of spermidine in lettuce response to excess of Zn, two additional investigations were carried out. As first, a Venn diagram (Figure 3) was generated starting from the differential metabolites resulting from Volcano analyses (Zn vs. control and NaCl vs. control, respectively). Indeed, Venn diagram evidenced that spermidine and S-adenosyl-L-homocysteine were exclusive of Zn-related differential metabolites. In the second confirmatory approach, the mathematical model underlying PLS-DA analysis was exported, and the model formula coefficient for each compound was recorded. Spermidine showed a coefficient of +0.146 and −0.512 on first and second vector, respectively. These values, despite confirming that polyamine response under Zn excess was not the univocal metabolic response to stress, still are in agreement with the distribution of Zn-related samples in the PLS-DA hyperspace as provided in Figure 2. Our findings are in agreement with previous work on the role of polyamines in alleviating the adverse effects of heavy metals stress (Luo et al., 2009; Aldesuquy et al., 2014). In addition to playing an important role in cell elongation and division in several plant species, polyamines are involved in the response to stress and their levels in plant increase under a number of environmental stress conditions such as drought, nutrient deficiency and phytotoxic metals (Aldesuquy et al., 2014). In several plants, polyamines can exist as free amines rather than being conjugated with hydroxycinnamic acids (hydroxycinnamic acid amides; Luo et al., 2009). Luo et al. (2009) reported that conjugated polyamines are accumulated in seeds and sometimes in roots, as a response to both biotic and abiotic stresses. A specific polyamine glucosyl transferase has been isolated (Luo et al., 2009), however its role in stress response is not fully elucidated and it has been postulated on the basis of correlative data. Recently, lettuce root pre-treatment with spermidine has been reported to enhance CO_2_ assimilation efficiency and to affect stomatal regulation (Sun et al., 2016). In the current experiment, polyamines have been specifically induced under Zn conditions, presumably to cope with the stress this metal induces to roots.

## Conclusions

Despite emerging as a diffuse soil contamination, not only linked to well-known point sources such as mining or steel industry, the stress from Zn excess to plant has not been deeply investigated yet. On the contrary, information on the effects of salinity to plants at molecular level is pretty available. In our work, lettuce root response to NaCl and zinc-induced stress was investigated at metabolome level under equimolar concentrations, to use elevate salinity as a “positive control.” The majority of differential roots metabolites were shared between the two stresses, although fold-changes were higher under NaCl stress condition. Most of the identified compounds could be ascribed to carbohydrates, phenolics, hormones, glucosinolates, and lipids. Our findings suggest that osmotic stress and redox imbalance play a major role in determining lettuce root response to NaCl and ZnSO_4_. However, accumulation of polyamines and polyamine conjugates has been specifically triggered by zinc.

The results provide insight into plant function under different environmental stimuli and are important in revealing the physiological/molecular mechanisms determining plant response to abiotic stresses, and to zinc exposure in particular. These findings at molecular level are useful to understand the biochemical processes that plant uses to cope with zinc stress. Our results might be also useful to assist the selection of tolerant cultivars and, possibly, be translated to other horticultural crops.

## Author contributions

LL designed the study, in cooperation with YR and MT. LL also coordinated the experimental work, developed the mass spectrometric method, performed statistics, and helped to draft the manuscript. LB carried out the plant experiments and metabolomic work, contributed to interpretation of data and drafted the manuscript. YR, GC MT, and DK participated in the interpretation of data, helped to draft the manuscript and to critically revise it for intellectual content. All authors read and approved the final manuscript, whereas LL was responsible of final approval of the version to be published.

### Conflict of interest statement

The authors declare that the research was conducted in the absence of any commercial or financial relationships that could be construed as a potential conflict of interest. The reviewer SS and handling Editor declared their shared affiliation, and the handling Editor states that the process nevertheless met the standards of a fair and objective review.
